# Is Red Heavier Than Yellow Even for Blind?

**DOI:** 10.1177/2041669518759123

**Published:** 2018-02-13

**Authors:** Marco Barilari, Adélaïde de Heering, Virginie Crollen, Olivier Collignon, Roberto Bottini

**Affiliations:** Center for Mind/Brain Sciences, University of Trento, Italy; International School for Advanced Studies, Trieste, Italy; Center for Research in Cognition & Neurosciences, Université Libre de Bruxelles, Belgium; Center for Mind/Brain Sciences, University of Trento, Italy; Institute of Psychology and Institute of Neuroscience, University of Louvain, Belgium; Center for Mind/Brain Sciences, University of Trento, Italy; Center for Mind/Brain Sciences, University of Trento, Italy; International School for Advanced Studies, Trieste, Italy

**Keywords:** Cross-modal correspondences, blindness, color, perceptual experience

## Abstract

Across cultures and languages, people find similarities between the products of different senses in mysterious ways. By studying what is called cross-modal correspondences, cognitive psychologists discovered that lemons are fast rather than slow, boulders are sour, and red is heavier than yellow. Are these cross-modal correspondences established via sensory perception or can they be learned merely through language? We contribute to this debate by demonstrating that early blind people who lack the perceptual experience of color also think that red is heavier than yellow but to a lesser extent than sighted do.

## Introduction

The majority of the population links apparently unrelated things in a way that is as amusing as it is mysterious. This is the case for cross-modal correspondences (CMCs).

CMC is a category of spontaneous and seemingly arbitrary associations between senses that are shared across cultures and languages (Spence, 2011). Perhaps the most popular example is the *bouba-kiki effect* according to which some auditory stimuli (e.g., kiki) sounds more *spiky* than others (e.g., bouba; Ramachandran & Hubbard, 2001). Several CMCs have been discovered over the years, and some of them revealed quite unexpected associations. For instance, most people think that lemons are *fast* rather than *slow* (Woods, Spence, Butcher, & Deroy, 2013), that boulders are *sour* rather than *sweet* (Brown, 1958), and that red is *heavier* than yellow (Alexander & Shansky, 1976). There is actually more in CMCs than first meets the eye since they may provide useful hints to understand mental phenomena such as synesthesia (when a stimulation in one sensory modality automatically activate experience in another sensory modality) and shed light on the evolution of language (Ramachandran & Hubbard, 2001).

Two different sets of hypothesis are currently battling. The *sensory hypothesis* suggests that CMCs are mild forms of synesthesia that rely on correspondences between low-level sensory processes (Ramachandran & Hubbard, 2001). In other words, our visuohaptic experience of boulders and our gustatory experience of sourness share some common neural codes that make them similar, across senses, as the taste of lemons and oranges is similar within the same sensory domain.

The *semantic hypothesis* instead suggests than CMCs reflect higher level (postsensory) semantic processes (Martino & Marks, 2001; Osgood, 1960; Walker & Walker, 2016). For instance, a heavy anonymous boulder may remind us the sourness of life rather than, let’s say, the sweetness of love.

Disentangling the two hypotheses is hard, especially because perceptual experience and semantic knowledge are usually correlated: We talk about what we perceive, and perceptual similarity can be encoded in language statistics (Louwerse, 2011). Here we seek to contribute to this debate by studying blind people who, compared to sighted individuals, have a different perceptual experience of the world while being exposed to similar language statistics. If CMCs are encoded in the semantic network that we use to think and communicate, it seems plausible that one could get these associations even though she or he cannot experience them with the senses. This translates to a simple experimental question: Is red heavier than yellow also for early blind people who don’t know what colors look like?

Ninety-two participants recruited in two different countries (70 Italians and 22 Belgians) completed a questionnaire in their native language (Italian and French, respectively): 46 early blind (EB) and 46 sighted controls (SC). Participants were pairwise matched between groups for gender (19 female), age (EB = 37.93, *SD* = 11.01; SC = 37.96, *SD* = 12.16), year of education (EB = 14.43, *SD* = 2.73 SC = 14.85, *SD* = 2.66), and native language. All EBs became totally blind before the age of 3 years and reported to have neither perception nor visual memory of colors.

Three questions with two alternative choices were orally presented to each participant. They were asked whether a lemon is fast or slow, whether a boulder is sour or sweet, and which one is heavier between red and yellow (in counterbalanced order).^1^ Crucially, the color-related item was the only one that could unambiguously provide information about the role of sensory experience, since color can only be perceived through sight. We therefore focused on the outputs of this question.

**Figure 1.**
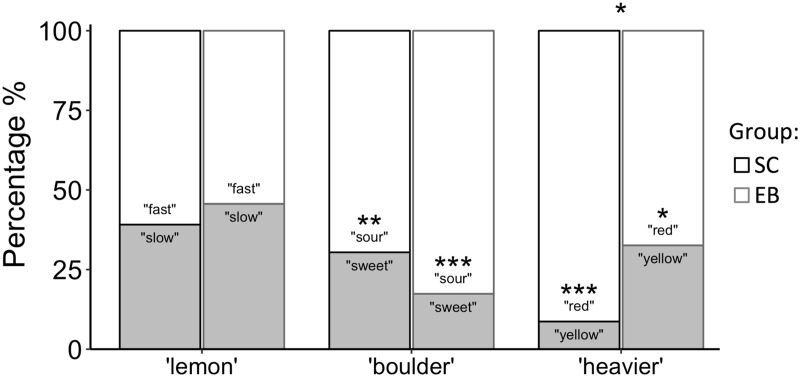
Bar graphs showing percentages of (binomial) alternatives choices for each question (e.g., boulder-sweet vs. boulder-sour) in sighted and blind. Asterisks indicate significant differences according to Chi-square tests (*p < .05, **p < .01, ***p < .001).

Chi-squared statistics were used to test whether participants chose an option (e.g., boulder-sour vs. boulder-sweet) above chance level, and whether this choice was different between groups. Results show that lemon was linked to the fast attribute in both groups, although not significantly so (SC: χ^2^(1) = 2.17, *p* = .140; EB: χ^2^(1) = .35, *p* = .555). A boulder was significantly *sour* both for sighted and blind (SC: χ^2^(1) = 7.04, *p* = .008; EB: χ^2^(1) = 19.56, *p* ≤ .001), and red was heavier than yellow for both groups (SC: χ^2^(1) = 31.40, *p* ≤ .001; EB: χ^2^(1) = 5.56, *p* = .018). Pairwise comparisons showed a significant difference across groups only for the color-related question (χ^2^(1) = 5.87, *p* = .015) (Figure 1).

Can CMCs be acquired without the relevant sensory experience? The answer is yes, since EB showed the same color-weight association of their sighted counterpart, therefore supporting the semantic hypothesis. However, we concomitantly find a significant difference across groups, as predicted by the *sensory hypothesis*: The bias to think that red was heavier than yellow was stronger in sighted than blind.^2^

Results suggest that CMCs such as the color-weight association are influenced by both linguistic and perceptual experience. We demonstrated indeed that such cross-modal correspondences are established even in the absence of the direct sensory experience (of colors, in this case) probably from linguistic patterns that encode semantic relationships. However, first-hand perceptual experience may strengthen cross-modal intuitions, in addition to semantic aspects.

## Notes

1 Initially, we also included a fourth question (Is a plum fast or slow?), but due to translation issues we had to exclude this item from the analysis.

2 Contrary to our predictions, the association lemon–fast did not reach significance, neither in sighted nor in blind. Yet, also in a previous report this CMC resulted to be weaker compared with the other two (Woods et al., 2013), and our study may lack adequate power to detect a relatively subtle effect. Indeed, pooling sighted and blind together (92 participants), the effect becomes marginal (χ^2^(1) = 2.13, *p* = .07, one-tailed). However, the strength of this CMC does not affect our conclusions.
